# Clinical characteristics and outcomes of immune-complex membranoproliferative glomerulonephritis and C3 glomerulopathy in Japanese children

**DOI:** 10.1007/s00467-024-06377-7

**Published:** 2024-04-25

**Authors:** Chika Ueda, Tomoko Horinouchi, Yuta Inoki, Yuta Ichikawa, Yu Tanaka, Hideaki Kitakado, Atsushi Kondo, Nana Sakakibara, China Nagano, Tomohiko Yamamura, Junya Fujimura, Naohiro Kamiyoshi, Shingo Ishimori, Takeshi Ninchoji, Hiroshi Kaito, Yuko Shima, Kazumoto Iijima, Kandai Nozu, Norishige Yoshikawa

**Affiliations:** 1 Department of Pediatrics, Kobe University Graduate School of Medicine, 7-5-1 Kusunoki-Cho, Chuo-Ku, Kobe, 650-0017 Japan; 2 Department of Pediatrics, Kakogawa Central City Hospital, 439 Honmachi, Kakogawa-Cho, Kakogawa, 675-8611 Japan; 3 Department of Pediatrics, Japanese Red Cross Society Himeji Hospital, 1-12-1 Shimoteno, Himeji, 670-8540 Japan; 4 Department of Pediatrics, Takatsuki General Hospital, 1-3-13 Kosobe‑cho, Takatsuki, 569-1192 Japan; 5 Department of Pediatrics, Harima-Himeji General Medical Center, 3-264 Kamiyacho, Himeji, 670-8560 Japan; 6 Department of Nephrology, Hyogo Prefectural Kobe Children’s Hospital, 1-6-7, Minatojima-Minamimachi, Chuo-Ku, Kobe, 650-0047 Japan; 7 Department of Pediatrics, Wakayama Medical University, 811-1 Kimiidera, Wakayama, 641-8509 Japan; 8 Hyogo Prefectural Kobe Children’s Hospital, 1-6-7, Minatojima-Minamimachi, Chuo-Ku, Kobe, 650-0047 Japan; 9 Department of Advanced Pediatric Medicine, Kobe University Graduate School of Medicine, 7-5-1, Kusunoki-Cho, Chuo-Ku, Kobe, 650-0017 Japan; 10 Clinical Research Center, Takatsuki General Hospital, 1-3-13 Kosobe-Cho, Takatsuki, 569-1192 Japan

**Keywords:** Membranoproliferative glomerulonephritis, Immune-complex MPGN, C3 glomerulopathy, C3 glomerulonephritis, Dense deposit disease

## Abstract

**Background:**

Membranoproliferative glomerulonephritis (MPGN) can be divided into immune-complex MPGN (IC-MPGN) and C3 glomerulopathy (C3G), which includes dense deposit disease (DDD) and C3 glomerulonephritis (C3GN). These conditions result from abnormalities in different complement pathways and may lead to different prognoses. However, there are limited studies describing the respective clinical courses.

**Methods:**

In this study, Japanese pediatric patients diagnosed with MPGN based on kidney biopsies conducted between February 2002 and December 2022 were reclassified as having IC-MPGN or C3G (DDD or C3GN). We retrospectively analyzed the clinical characteristics and outcomes of these patients.

**Results:**

Out of 25 patients with MPGN, three (12.0%) were diagnosed with DDD, 20 (80.0%) with C3GN, and two (8.0%) with IC-MPGN. There were 13 (65.0%) patients and one (33.3%) patient in remission after treatment for C3GN and DDD, respectively, and no patients with IC-MPGN achieved remission. The median follow-up period was 5.3 (2.5–8.9) years, and none of the patients in either group progressed to an estimated glomerular filtration rate < 15 ml/min/1.73 m^2^. Patients with C3GN presenting mild to moderate proteinuria (*n* = 8) received a renin-angiotensin system inhibitor (RAS-I) alone, and these patients exhibited a significant decrease in the urinary protein creatinine ratio and a notable increase in serum C3 levels at the last follow-up.

**Conclusions:**

Most patients with MPGN were diagnosed with C3GN. The remission rate for C3GN was high, and no patients developed kidney failure during the approximately 5-year follow-up. Additionally, patients with C3GN with mild to moderate proteinuria had good outcomes with RAS-I alone, but continued vigilance is necessary to determine long-term prognosis.

**Graphical abstract:**

**Figure:**
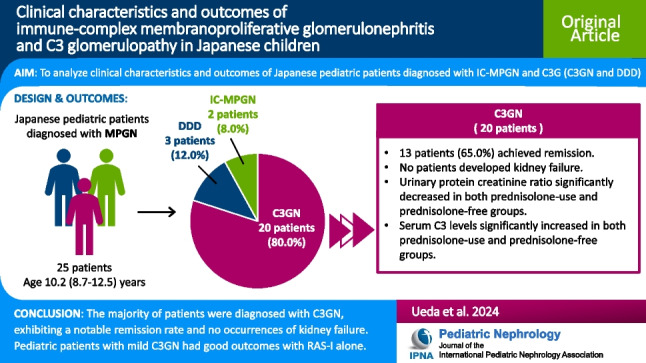
A higher resolution version of the Graphical abstract is available as Supplementary information

**Supplementary Information:**

The online version contains supplementary material available at 10.1007/s00467-024-06377-7.

## Introduction

Membranoproliferative glomerulonephritis (MPGN) is a glomerular injury characterized by pathological findings of mesangial interposition and double contours of the capillary wall [1]. Historically, MPGN was morphologically classified into three subtypes based on the deposition site of electron-dense deposits (EDD) in the glomerulus, as observed by electron microscopy: type I with EDD in the subendothelial areas, type II with deposits in the basement membrane, and type III with deposits in both the subepithelial and subendothelial areas [2, 3]. Then, it was determined that type II was caused by congenital abnormalities in the alternative pathway, leading to the establishment of a distinct disease concept called dense deposit disease (DDD) [4]. However, in a 2012 study, immunofluorescence images revealed strong positivity for C3 deposits in DDD. On the other hand, in type I and III, cases with strong C3 deposition similar to DDD and cases with both C3 and immunoglobulin deposition coexisted, indicating potentially different etiologies [5]. Subsequently, based on immunofluorescence images, patients with predominant IgG staining were reclassified as having immune-complex MPGN (IC-MPGN), while those with predominant C3 deposition were reclassified as having C3 glomerulopathy (C3G) [5, 6]. C3G was further classified into DDD and C3 glomerulonephritis (C3GN) with DDD characterized by linear intramembranous EDD according to electron microscopy, and C3GN was classified when there was absence of DDD [5, 6]. The diagnosis of C3G is based on immunofluorescence findings rather than light microscopy, so glomerulonephritis patterns such as mesangial proliferative, crescentic and acute proliferative and exudative patterns are now included, although the primary histological feature is MPGN. IC-MPGN is caused by activation of the classical pathway due to immune complex formation [5]. On the other hand, C3G (DDD, C3GN) is caused by the dysregulation of alternative pathway activity [5, 7]. Given the different complement pathways involved, IC-MPGN and C3G (DDD, C3GN) may exhibit distinct prognoses. However, limited reports exist on the respective clinical courses of IC-MPGN and C3G. There is also a lack of evidence-based standard treatment for patients with IC-MPGN and C3G, particularly in the pediatric population [8]. Furthermore, IC-MPGN is more commonly secondary to causes such as infections, autoimmune diseases, and monoclonal gammopathies, further decreasing its prevalence when excluding these factors.

The objective of this study was to determine the distribution of the new classification in patients previously diagnosed with MPGN, and furthermore, to clarify the clinical characteristics of IC-MPGN, DDD, and C3GN individually. To achieve this, we reclassified types of MPGN based on kidney biopsy findings in pediatric patients diagnosed with MPGN. Subsequently, we retrospectively analyzed demographic, clinical and laboratory findings, and therapeutic response in patients with IC-MPGN and C3G (DDD, C3GN).

## Methods

### Participants and study design

This retrospective cohort study included Japanese pediatric patients diagnosed with MPGN via kidney biopsy between February 2002 and December 2022 at the pediatric nephrology departments of Kobe University Hospital, Wakayama Medical University Hospital, Hyogo Prefectural Kobe Children’s Hospital, Takatsuki General Hospital, Japanese Red Cross Society Himeji Hospital, and Kakogawa Central City Hospital. The exclusion criteria were patients with missing clinical or laboratory data, absence of immunofluorescence imaging, resolved proteinuria without therapeutic intervention, and a brief follow-up period (< 6 months). We also excluded patients indicated to have secondary nephritis and included only those with idiopathic MPGN. The following data were retrospectively obtained from the patients’ medical records at the time of diagnosis (initial kidney biopsy) and subsequent regular outpatient visits, as well as at the last follow-up: patient demographic information (age, sex, height, and weight), clinical and laboratory data (proteinuria, hematuria, serum creatinine level, estimated glomerular filtration rate (eGFR), serum albumin level, serum C3 and C4 level and presence of nephrotic syndrome), and details of the treatment. For C3GN, the predominant subtype of MPGN, we examined the factors associated with remission and normalization of serum C3 levels. In addition, we identified clinical and laboratory findings with and without prednisolone. The Kaplan‒Meier method was used to assess the time to remission or normalization of the serum C3 levels.

### Pathology

Kidney biopsies were performed for children exhibiting both hypocomplementemia and proteinuria. All kidney biopsy reports, including light microscopy, immunostaining, and electron microscopy results, were reviewed by one pathologist (NY). MPGN was pathologically diagnosed based on mesangial hypercellularity, endocapillary hypercellularity, and double-contour formation along the glomerular capillary walls via light microscopy. Subsequently, based on immunofluorescence imaging of kidney biopsies, we reclassified these patients with MPGN as having IC-MPGN or C3G. C3-dominant deposits were categorized as C3G with ‘dominant’, defined as a C3 intensity two orders of magnitude higher than that of any other immune reactant (IgG, IgA, IgM or C1q) on a scale of zero to three [9], and the others were categorized as having IC-MPGN [5, 6]. Further subclassification of C3G included DDD or C3GN: DDD was assigned when linear intramembranous EDD were observed via electron microscopy, while those lacking DDD characteristics were classified as having C3GN [5, 6]. In patients with multiple kidney biopsies, the initial biopsy was used for diagnosis.

### Treatment protocol

Treatments were administered at the discretion of the clinicians. In general, patients exhibiting mild to moderate proteinuria (urine protein creatinine ratio 0.15–1.0 g/gCr) were treated with angiotensin-converting enzyme inhibitor (ACE-I), patients who did not respond adequately to ACE-I were additionally given an angiotensin II receptor blocker (ARB). Those facing challenges in continuing ACE-I medication due to side effects such as cough and dizziness were switched to an ARB. Patients diagnosed with nephrotic syndrome or severe proteinuria (urinary protein creatinine ratio ≥ 1.0 g/gCr) or those experiencing exacerbation after ACE-I or ARB treatment received either prednisolone or a combination of prednisolone and intravenous methylprednisolone at a dosage of 30 mg/kg/day (maximum dose, 1000 mg/day) for three consecutive days. This regimen is known as methylprednisolone pulse therapy (MPT) and was administered for two to three courses along with prednisolone. In cases of worsening or inadequate response, an additional two courses of MPT were administered, followed by prednisolone, and subsequently, immunosuppressants such as mizoribine (MZR) or mycophenolate mofetil (MMF) were added.

### Definitions

Nephrotic syndrome was diagnosed based on the presence of both severe proteinuria (urine protein creatinine ratio ≥ 2 g/gCr) and hypoalbuminemia (serum albumin ≤ 2.5 g/dL). Hematuria was defined as the presence of ≥ 5 red blood cells per high-power field of a centrifuged urine specimen. Proteinuria was defined as a protein creatinine ratio ≥ 0.15 g/gCr. Remission was considered to be achieved when the protein creatinine ratio was < 0.15 g/gCr and the serum albumin levels were > 2.5 g/dL. Normalization of the serum C3 levels was considered to be achieved when the serum C3 levels were > 80 mg/dL. For patients aged between two and eighteen years, the eGFR was calculated by comparing the serum creatinine levels of patients with the reference serum creatinine levels of Japanese children based on the patients’ height and sex [10]. For patients aged > 18 years, the eGFR was calculated based on the patients’ serum creatinine levels, age, and sex [11].

### Statistical methods

Statistical analysis was conducted using Easy R (EZR) [12], a modified version of the R Commander that is specifically tailored to include commonly-used statistical methods in biostatistics. Clinical and laboratory findings are presented as the median and interquartile range (IQR) for continuous variables and as percentages for qualitative variables. The Mann‒Whitney U test and Wilcoxon signed rank test were employed to assess differences between continuous variables, while Fisher's exact test and McNemar's test were utilized for comparing qualitative variables. The Kaplan–Meier method was used to evaluate the time to remission and serum C3 normalization among patients. *P* values < 0.05 were considered to indicate statistical significance.

## Results

### Study cohort

**Fig. 1:**
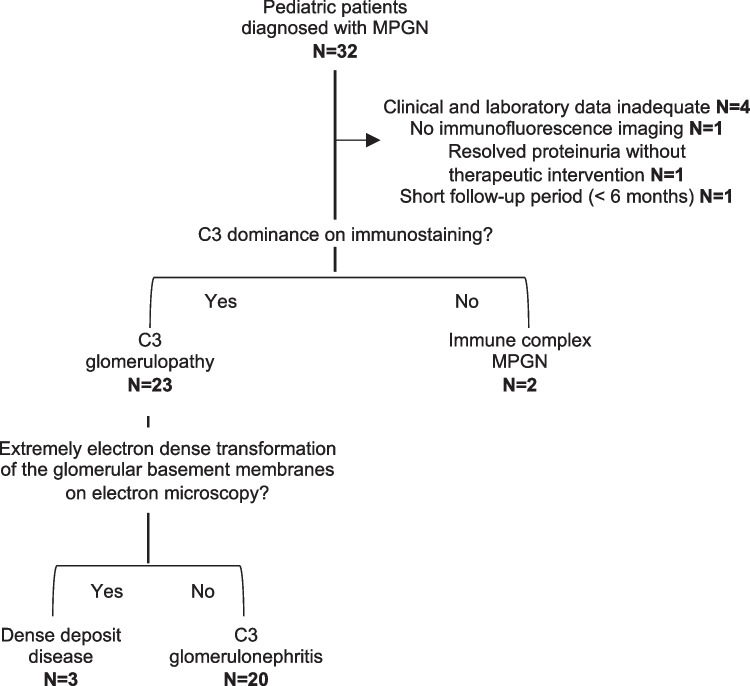
Flow chart showing the distribution of pediatric patients with MPGN. This study included 32 pediatric patients diagnosed with MPGN based on kidney biopsies conducted between February 2002 and December 2022. Patients with inadequate clinical and laboratory data (*n*=4), no immunofluorescence imaging (n=1), resolved proteinuria without therapeutic intervention (*n*=1), and short follow-up (< 6 months) (*n*=1) were excluded from the analysis. Of the remaining patients, 23 with C3 dominant deposits were classified as C3G, and the other two patients were classified as IC-MPGN. Furthermore, among the patients with C3G, three patients exhibiting linear intramembranous electron dense deposits on electron microscopy were classified as DDD, while the remaining 20 patients were classified as having C3GN.

In this retrospective cohort study, 32 patients were diagnosed with idiopathic MPGN. We excluded seven patients: four patients due to inadequate clinical and laboratory data, one patient with no immunofluorescence imaging, one patient with resolved proteinuria without therapeutic intervention, and one patient with a short follow-up period (< 6 months). There were no cases of suspected secondary MPGN. Among the remaining 25 patients with MPGN, three (12.0%) were reclassified as DDD, 20 (80.0%) were reclassified as C3GN, and two (8.0%) were reclassified as IC-MPGN (Fig. 1).

### Patient demographics and clinical characteristics

The events that led to the diagnosis of MPGN in 25 patients were as follows: school urinalysis in 16 patients, abnormal findings in a urine test conducted incidentally in five patients, onset of nephrotic syndrome in two patients, and gross hematuria in two patients. The baseline clinical characteristics are shown in Table 1. The median age at diagnosis for the entire cohort was 10.2 (8.7–12.5) years, and 16 (64.0%) patients were male. Hematuria was observed in 24 (96.0%) patients. Among patients with C3GN, only two (10.0%) presented with nephrotic syndrome, while no patients with DDD presented nephrotic syndrome. Conversely, both patients with IC-MPGN exhibited nephrotic syndrome. The median urine protein creatinine ratio at diagnosis was 1.14 (0.47–1.72) g/gCr. The median eGFR for the entire cohort was 122.5 (103.1–137.6) mL/min/1.73 m^2^, with five (20.0%) patients showing an eGFR < 90 ml/min/1.73 m^2^. The median serum C3 level was 18.0 (12.0–31.5) mg/dL, and the serum C3 levels were low at the time of diagnosis in all groups.

**Table 1:** Baseline clinical characteristics in patients with MPGN

	Total (*N* = 25)	C3G		IC-MPGN (*N* = 2)
		C3GN (*N* = 20)	DDD (*N* = 3)	
Age at diagnosis (year)	10.2 (8.7–12.5)	9.8 (8.6–13.0)	10.6 (9.6–11.2)	10.1 (9.4–10.9)
Male, *N* (%)	16 (64.0)	14 (70.0)	1 (33.3)	1 (50.0)
Height (cm)	132.2 (126.2–156.6)	134.4 (126.2–159.1)	132.2 (131.6–139.1)	134.1 (129.1–139.2)
Weight (kg)	28.1 (24.0–45.9)	27.9 (24.0–50.1)	28.1 (27.3–30.3)	27.9 (25.4–30.5)
Hematuria, *N* (%)	24 (96.0)	19 (95.0)	3 (100)	2 (100)
Nephrotic syndrome, *N* (%)	4 (16.0)	2 (10.0)	0 (0)	2 (100)
Urine protein creatinine ratio (g/gCr)	1.14* (0.47–1.72)	0.86 (0.42–1.28)	2.86 (1.96–4.28)	4.50*
eGFR (ml/min/1.73 m^2^)	122.5 (103.1–137.6)	124.2 (105.2–139.3)	122.5 (101.3–124.5)	182.7 (140.6–224.8)
eGFR < 90 ml/min/1.73 m^2^, *N* (%)	5 (20.0)	4 (20.0)	1 (33.3)	0 (0)
Serum albumin (g/dL)	4.0 (3.6–4.2)	4.0 (3.7–4.2)	4.1 (3.9–4.4)	2.3 (2.3–2.4)
Serum C3 (mg/dL)	18.0* (12.0–31.5)	18.0 (12.0–31.8)	12.0 (8.6–16.5)	33.0*
Serum C4 (mg/dL)	15.1* (11.8–18.2)	16.0 (12.8–19.0)	11.0 (10.0–12.0)	7.0*
*Lack of data for 1 patient				

*MPGN*, membranoproliferative glomerulonephritis; *C3G*, C3 glomerulopathy; *C3GN*, C3 glomerulonephritis; *DDD*, dense deposit disease; *IC-MPGN*, immune-complex MPGN; *eGFR*, estimated glomerular filtration rate

### Pathological findings

The light microscopic findings are shown in Table 2. Notably, crescents, global sclerosis and interstitial fibrosis were uncommon among our patients. Crescents were observed in five (25.0%) patients with C3GN, one (33.3%) patient with DDD, and one (50.0%) patient with IC-MPGN. Global sclerosis was present in one (5.0%) patient with C3GN, one (33.3%) patient with DDD, and one (50.0%) patient with IC-MPGN. Interstitial fibrosis was absent in patients with DDD but was observed in six (30.0%) patients with C3GN and one (50.0%) patient with IC-MPGN.

**Table 2:** Light microscopic findings in patients with MPGN

	C3G		IC-MPGN (*N* = 2)
	C3GN (*N* = 20)	DDD (*N* = 3)	
Crescents, N (%)			
None	15 (75.0)	2 (66.7)	1 (50.0)
< 50% glomeruli	5 (25.0)	1 (33.3)	1 (50.0)
≥ 50% glomeruli	0 (0)	0 (0)	0 (0)
Endocapillary hypercellularity, N (%)	3 (15.0)	2 (66.7)	0 (0)
Global sclerosis, N (%)	1 (5.0)	1 (33.3)	1 (50.0)
Interstitial fibrosis, N (%)	6 (30.0)	0 (0)	1 (50.0)

*MPGN*, membranoproliferative glomerulonephritis; *C3G*, C3 glomerulopathy; *C3GN*, C3 glomerulonephritis; *DDD*, dense deposit disease; *IC-MPGN*, immune-complex MPGN

### Treatments

As shown in Table 3, 23 (92.0%) patients received RAS-I. Prednisolone was administered to all patients with DDD and IC-MPGN, but to just 12 (60.0%) patients with C3GN. Immunosuppressants were administered to all patients with IC-MPGN and to four patients (20.0%) with C3GN, but not to patients with DDD. Based on the clinicians’ decisions, some patients received treatments that deviated from the strategy described in the Methods section. Specifically, one patient with C3GN and mild proteinuria was treated with prednisolone and MMF in addition to ACE-I. Another patient with C3GN and with initially mild proteinuria received ACE-I, but prednisolone and MMF were added due to an inadequate response. Subsequently, due to exacerbation, three courses of MPT were administered, followed by prednisolone. One patient with IC-MPGN with severe proteinuria received cyclosporine instead of MZR or MMF.

**Table 3:** Treatments and outcomes in patients with MPGN

	Total (*N* = 25)	C3G (*N* = 23)		IC-MPGN (*N* = 2)
		C3GN (*N* = 20)	DDD (*N* = 3)	
Therapy during the follow-up period				
RAS-I, *N* (%)	23 (92.0)	18 (90.0)	3 (100)	2 (100)
Prednisolone, *N* (%)	17 (68.0)	12 (60.0)	3 (100)	2 (100)
Immunosuppressants, *N* (%)	6 (24.0)	4 (20.0)	0 (0)	2 (100.0)
Therapy at the last follow-up				
RAS-I, *N* (%)	15 (60.0)	11 (55.0)	2 (66.7)	2 (100)
Prednisolone, *N* (%)	6 (24.0)	4 (20.0)	2 (66.7)	0 (0)
Immunosuppressants, *N* (%)	5 (20.0)	4 (20.0)	0 (0)	1 (50.0)
Clinical parameters at the last follow-up				
Hematuria, *N* (%)	7 (28.0)	5 (25.0)	1 (33.3)	1 (50.0)
Urine protein creatinine ratio (g/gCr)	0.12 (0.06–0.25)	0.10 (0.05–0.23)	0.17 (0.13–0.31)	0.28 (0.24–0.31)
Remission, *N* (%)	14 (56.0)	13 (65.0)	1 (33.3)	0 (0)
eGFR (ml/min/1.73 m^2^)	110.1* (103.7–121.5)	107.8* (102.8–121.3)	113.3 (112.6–119.9)	99.7 (88.7–110.7)
eGFR < 90 ml/min/1.73 m^2^, *N* (%)	3* (12.0)	2* (10.0)	0 (0)	1 (50.0)
Serum albumin (g/dL)	4.3 (4.1–4.6)	4.3 (4.1–4.6)	4.6 (4.1–4.8)	4.3 (4.2–4.4)
Serum C3 (mg/dL)	76.0** (47.5–92.5)	72.0* (47.5–89.0)	46.0* (28.0–63.9)	97.5 (95.3–99.8)
Serum C4 (mg/dL)	16.0** (14.9–22.0)	16.0* (14.9–21.0)	14.6* (12.8–16.4)	24.0 (23.5–24.5)

*Lack of data for one patient. **Lack of data for two patients

*MPGN*, membranoproliferative glomerulonephritis; *C3G*, C3 glomerulopathy; *C3GN*, C3 glomerulonephritis; *DDD*, dense deposit disease; *IC-MPGN*, immune-complex MPGN; *RAS-I*, renin-angiotensin system inhibitor; *eGFR*, estimated glomerular filtration rate

### Outcome

The outcomes of this retrospective cohort study are shown in Table 3. The follow-up period of the entire cohort was 5.3 (2.5–8.9) years. The incidence of hematuria at the last follow-up decreased in all groups (28%) compared with that at diagnosis. Thirteen (65.0%) patients with C3GN and one (33.3%) patient with DDD achieved remission, but none of the patients with IC-MPGN achieved remission. No patients in either group progressed to an eGFR < 15 ml/min/1.73 m^2^. Notably, compared with those at the time of diagnosis, urinary protein at the last follow-up tended to decrease, and serum C3 levels tended to increase in all groups (Supplementary Fig. 1).

Clinical and pathological findings as well as treatments of patients with C3GN in remission and non-remission are shown in Table 4. Baseline clinical findings and treatments did not differ between the remission and non-remission groups. Pathology revealed that, in the non-remission group, no patients had crescents, endocapillary hypercellularity, or global sclerosis, and only one (14.3%) patient had interstitial fibrosis. Clinical and pathological findings and treatments for patients with C3GN with normalized and non-normalized serum C3 levels are shown in Supplementary Table 1. The age at diagnosis was significantly lower in patients with normalized serum C3 levels than in those with non-normalized serum C3 levels (8.7 vs. 12.7 years, *P* = 0.02). Additionally, patients with normalized serum C3 levels had significantly lower urine protein creatinine ratio at the last follow-up (0.04 vs. 0.17 g/gCr, *P* = 0.02) and a significantly higher remission rate (100.0% vs. 50.0%, *P* = 0.04) compared with those without normalization. There were no significant differences in other clinical findings, pathological findings, or treatments between the patients with normalized and non-normalized serum C3 levels.

**Table 4:** Characteristics of remission and non-remission cases in the C3GN group

	Remission (*N* = 13)	Non-remission (*N* = 7)	*P* value
Baseline			
Age at diagnosis (year)	8.9 (8.2–12.3)	12.8 (10.7–13.8)	0.14
Nephrotic syndrome, *N* (%)	2 (15.4)	0 (0)	0.52
eGFR (ml/min/1.73 m^2^)	126.7 (109.2–144.2)	122.4 (104.5–130.6)	0.54
eGFR < 90 ml/min/1.73 m^2^, *N* (%)	3 (23.1)	1 (14.3)	1
Urinary protein creatine ratio (g/gCr)	0.71 (0.48–1.29)	1.21 (0.34–1.27)	0.66
Serum C3 (mg/dL)	19.0 (15.0–34.0)	16.0 (7.5–30.5)	0.48
Low C3 (≤ 80 mg/dL), *N* (%)	13 (100.0)	6 (85.7)	0.35
Histopathology			
Crescents, *N* (%)	5 (38.5)	0 (0)	0.11
Endocapillary hypercellularity, *N* (%)	3 (23.1)	0 (0)	0.52
Global sclerosis, *N* (%)	1 (7.7)	0 (0)	1
Interstitial fibrosis, *N* (%)	5 (38.5)	1 (14.3)	0.35
Treatment			
RAS-I, *N* (%)	11 (84.6)	7 (100)	0.52
Prednisolone, *N* (%)	7 (53.8)	5 (71.4)	0.64
MZR, MMF, *N* (%)	1 (7.7)	3 (42.9)	0.1

*C3GN*, C3 glomerulonephritis; *eGFR*, estimated glomerular filtration rate; *RAS-I*, renin-angiotensin system inhibitor; *MZR*, mizoribine; *MMF*, mycophenolate mofetil

Initial biopsy findings and clinical outcomes, stratified by need for prednisolone at diagnosis in the C3GN group, are shown in Table 5. Hematuria, eGFR, urine protein creatinine ratio, and serum C3 levels were compared between the time of diagnosis and the time of the last follow-up within each group. In both groups, the urinary protein creatinine ratio significantly decreased and the serum C3 levels significantly increased at the last follow-up. Remission was achieved in 6 of the 8 (75.0%) patients in the prednisolone-free group and 7 of the 12 (58.3%) patients in the group in which prednisolone was used. Among the four patients with severe proteinuria who received MZR or MMF, only one achieved remission, and none of them had normalized serum C3 levels.

**Table 5:** Initial biopsy findings and clinical outcomes, stratified by need for prednisolone in C3GN

	Prednisolone-free (*N* = 8)	Prednisolone-used (*N* = 12)
Histopathology		
Crescents at diagnosis, *N* (%)	3 (37.5)	2 (16.7)
Endocapillary hypercellularity at diagnosis, *N* (%)	1 (12.5)	2 (16.7)
Global sclerosis at diagnosis, *N* (%)	0 (0)	1 (8.3)
Interstitial fibrosis at diagnosis, *N* (%)	1 (12.5)	5 (41.7)
Clinical variables		
Hematuria at diagnosis, *N* (%)	8 (100)	11 (91.7)
Hematuria at the last follow-up, *N* (%)	1 (12.5)	4 (33.3)
* P* value	NA	0.02
eGFR at diagnosis (ml/min/1.73 m^2^)	126.8 (104.1–135.9)	121.6 (105.2–144.7)
eGFR at the last follow-up (ml/min/1.73 m^2^)	110.7 (106.1–126.6)	106.3* (100.6–121.3)
* P* value	0.84	0.12
eGFR < 90 ml/min/1.73 m^2^ at diagnosis, *N* (%)	2 (25.0)	2 (16.7)
eGFR < 90 ml/min/1.73 m^2^ at the last follow-up, *N* (%)	1 (12.5)	1* (9.1)
* P* value	1	1
Urine protein creatinine ratio at diagnosis (g/gCr)	0.54 (0.43–0.79)	1.27 (0.42–1.72)
Urine protein creatinine ratio at the last follow-up (g/gCr)	0.09 (0.06–0.15)	0.10 (0.05–0.43)
* P* value	0.02	0.03
Remission, *N* (%)	6 (75.0)	7 (58.3)
Serum C3 (mg/dL) at diagnosis	18.0 (15.8–31.5)	15.5 (10.5–31.8)
Serum C3 (mg/dL) at the last follow-up	70.5 (64.3–88.8)	76.0^*^ (28.0–88.5)
* P* value	0.02	0.004
Normalization of serum C3, *N* (%)	3 (37.5)	4* (36.4)
*Lack of data for one patient		

*C3GN*, C3 glomerulonephritis; *eGFR*, estimated glomerular filtration rate; *NA*, not available

**Fig. 2:**
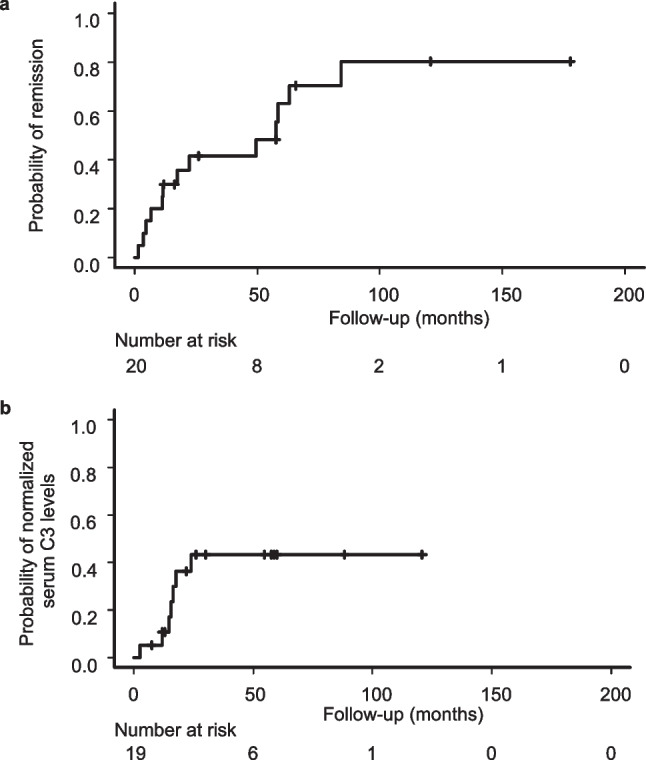
Kaplan–Meier analysis of remission by the last follow-up and normalized serum C3 levels in patients with C3GN. (**a**) The median time to remission by the last follow-up was 53.3 months. (**b**) The median time to normalized serum C3 levels was not available because the rate of normalization did not reach 50%

A Kaplan–Meier analysis of remission and normalization of serum C3 levels in patients with C3GN is shown in Fig. 2. The median time to remission was 53.3 months. The median time to normalized serum C3 levels was not available because the rate of normalization did not reach 50%.

## Discussion

This retrospective cohort study reports the comprehensive clinical and laboratory data and outcomes of pediatric patients diagnosed with IC-MPGN and C3G (DDD, C3GN) at several pediatric facilities in Japan.

Surprisingly, of the 25 patients diagnosed with MPGN in this study, 20 (80.0%) patients were classified as having C3GN. The percentage of C3GN in MPGN was previously reported as 29.5–35.7% in pediatric cohorts [13, 14], 37.1–41.7% in mixed pediatric and adult cohorts [15, 16], and 39.3% in an adult cohort [17]. The proportion of C3GN among the patients with MPGN in this study was thus greater than that in previous reports. Due to the small number of patients diagnosed with DDD and IC-MPGN, we examined the clinical characteristics and outcomes of patients with C3GN in this study.

In this study, the median age at diagnosis for patients with C3GN was 9.8 (8.6–13.0) years, which is like that reported in other pediatric reports (9.0–10.1 years) [7, 13, 14]. Furthermore, in this study, the prevalence of hematuria in patients with C3GN was 95.0%, which was present in most patients and comparable to previous reports of children and adults [13–15]. In patients with MPGN, the presence of nephrotic syndrome [18] and decreased eGFR at diagnosis [19] are considered to be poor prognostic signs. In previous reports, the complication rate of nephrotic syndrome in patients with C3GN ranged from 26.8–32.6% in mixed cohorts of children and adults [15, 16, 19] to 76.9% in a pediatric cohort [13]. However, in this study, the prevalence was 10.0%, which is clearly lower than that in the previous reports. Regarding kidney function, the eGFR at baseline in this study was 124.2 (105.2–139.3) ml/min/1.73 m^2^ and the percentage of eGFR < 90 ml/min/1.73 m^2^ was 20.0%. This indicates good kidney function, compared with previous reports of 65.9–75.7 ml/min/1.73 m^2^ in mixed cohorts of children and adults [16, 19], and 101 ml/min/1.73 m^2^ [13] and 44.0% of eGFR < 90 ml/min/1.73 m^2^ [14] in children. Regarding the pathological findings of C3GN, the presence of crescents [20–23], tubulointerstitial disease [22, 23] and sclerosis [21] are considered to be poor prognostic signs in patients with MPGN. In this study, 75.0% of the patients with C3GN had no crescents, 25.0% had less than 50% crescents, and none had 50% or more crescents. In addition, 30.0% of patients with C3GN had interstitial fibrosis. The complication rate of these findings was comparable to that of previous pediatric cohorts [14]. The complication rate of global sclerosis was 5.0% in this study, which was lower than that in previous pediatric cohorts [13, 14]. Our Japanese cohort can be said to be predominantly composed of patients with mild symptoms overall. On the other hand, the serum C3 level in this study was 18.0 (12.0–31.5) mg/dL, which was relatively low compared with previous reports of 27.5–39.0 mg/dL in a pediatric cohort [13, 14] and 44.0 mg/dL in a mixed cohort of adults and children [15].

C3GN is often reported as refractory. However, under the definition of remission as a protein creatinine ratio < 0.15 g/gCr and serum albumin levels > 2.5 g/dL, remission occurred in 13 of the 20 (65.0%) patients with C3GN. Previous papers [13, 14, 19] have reported remission rates of 11.5–60.0% for patients with C3GN, and although the definition of remission in this study was stricter than those previously reported, the remission rate was similar or greater. In addition, in previous reports [13, 19], 7.7–9.2% of patients with C3GN progressed to an eGFR < 15 ml/min/1.73 m^2^ at the last follow-up, compared with no patients in this study. The patients with C3GN in this study had a lower complication rate of nephrotic syndrome and better kidney function and pathological findings of the kidneys, suggesting a greater proportion of mild cases than previously reported. In Japan, a school urinalysis system is widespread, and regular urinalysis allows early diagnosis of C3GN. This may have resulted in a greater percentage of mild cases in this study than in previous reports that were based outside of Japan. In fact, 14 of the 20 patients with C3GN in our study were diagnosed following an abnormality detected in a school urinalysis. Another study of eight Japanese pediatric cases of C3GN [24] reported that none of their patients progressed to an eGFR < 15 ml/min/1.73 m^2^ at the last follow-up. Additionally, the median eGFR at diagnosis was 127.8 ml/min/1.73 m^2^, indicating good kidney function, like our study.

Interestingly, the remission rate was lower (12.5%) in their study compared to our study (65.0%). This could be attributed to the stricter definition of remission than ours. Additionally, the difference in the definition in C3GN, which was defined as deposition of C3 alone in the previous study, may also contribute to the lower remission rate of C3GN compared to our study.

In this study, the prednisolone-free group exhibited a significant decrease in urinary protein and a significant increase in serum C3 at the last follow-up. In addition, remission was achieved in six of the eight (75.0%) patients, suggesting a good response to treatment. In a previous report [14], all seven pediatric patients with C3GN treated with ACE-I or ARB alone also achieved remission. In contrast, cohorts of adults with C3G [25, 26] reported that 67–79% of patients treated with MMF and steroids achieved at least partial remission, with only 10–14% progressing to kidney failure, indicating a favorable outcome. None of the patients in the prednisolone-free group in this study had nephrotic syndrome, with an eGFR of 126.8 ml/min/1.73 m^2^ and a urinary protein creatinine ratio of 0.54 g/gCr at diagnosis. In the adult cohort, 30–43% of patients with C3G had nephrotic syndrome, with an eGFR of 57–67 ml/min/1.73 m^2^ and a urinary protein creatinine ratio of 2.47–3.00 g/gCr. The differences in the severity of included patients may be contributing to variations in prognosis. In the current study, the group in which prednisolone was used also had a significant decrease in urinary protein and a significant increase in serum C3 levels at the last follow-up, suggesting that even patients with C3GN with nephrotic syndrome or severe proteinuria (urinary protein creatinine ratio ≥ 1.0 g/gCr) may have favorable outcomes with prednisolone. However, there was no statistically significant difference in urinary protein and serum C3 levels between pre- and post-treatment in patients treated with MZR or MMF. These immunosuppressants were added when patients had worsening or inadequate response to prednisolone. Treatment response is therefore limited in such severe cases in which immunosuppressants are needed.

When discussing the percentage of patients with C3GN and evaluating its severity and outcomes, the presence of post-infectious glomerulonephritis should be considered. Specifically, cases of post-infectious glomerulonephritis beyond the acute phase may exhibit C3 deposition without immunoglobulin [27]. Conversely, C3GN may also be present after an infectious episode, and the presence of subepithelial humps commonly observed in post-infectious glomerulonephritis often characterizes C3GN [9]. Thus, it is often difficult to distinguish between the two diseases. In this study, some cases were discovered asymptomatically through urine screening. We cannot deny the possibility that these asymptomatic cases had subclinical acute post-infectious glomerulonephritis, potentially contributing to the favorable prognosis. On the other hand, according to consensus reports on C3G [9], serum C3 levels normalize within eight to 12 weeks in typical post-infectious glomerulonephritis cases. In this study, all of patients with C3GN exhibited low serum C3 levels for more than 12 weeks. Therefore, at least the typical post-infectious glomerulonephritis cases were not included in this study.

This study has several limitations. First, the investigation was retrospective, and we did not include a control group to compare outcomes with those of other treatments. Second, all patients in this study were Japanese children. Third, we could not obtain information on autoantibodies and genetic variants related to the alternative pathway of the complement system. Fourth, because the sample sizes of patients with DDD and IC-MPGN were relatively small, it was not possible to compare properly the clinical characteristics of the two diseases. However, the study included a relatively large number of patients despite the rarity of the disease. In addition, the diagnosis and classification of the MPGNs were performed by a single pathologist, which ensured consistency in the diagnosis.

In summary, most pediatric patients with MPGN were diagnosed with C3GN. Overall, 65.0% of patients with C3GN achieved remission, and none progressed to an eGFR < 15 ml/min/1.73 m^2^ at the last follow-up, indicating good outcomes. In particular, patients with C3GN with mild to moderate proteinuria had good outcomes with RAS-I alone.

### Supplementary Information

Below is the link to the electronic supplementary material.

Graphical abstract (PPTX 96.0 KB)

Supplementary file2 (DOCX 153 KB)

## Data Availability

Data from this study can be obtained from the corresponding author upon reasonable request.
