# Supplementary material for: Clinical characteristics and outcomes of immune-complex membranoproliferative glomerulonephritis and C3 glomerulopathy in Japanese children
Source: Pediatr Nephrol. 2024 Apr 25;39(9):2679–89. doi: 10.1007/s00467-024-06377-7 (PMC11272671; doi:10.1007/s00467-024-06377-7)
Supplement: Supplementary file 1 — Graphical abstract (PPTX 96.0 KB) [file 467_2024_6377_MOESM1_ESM.pptx]

## Slide 1
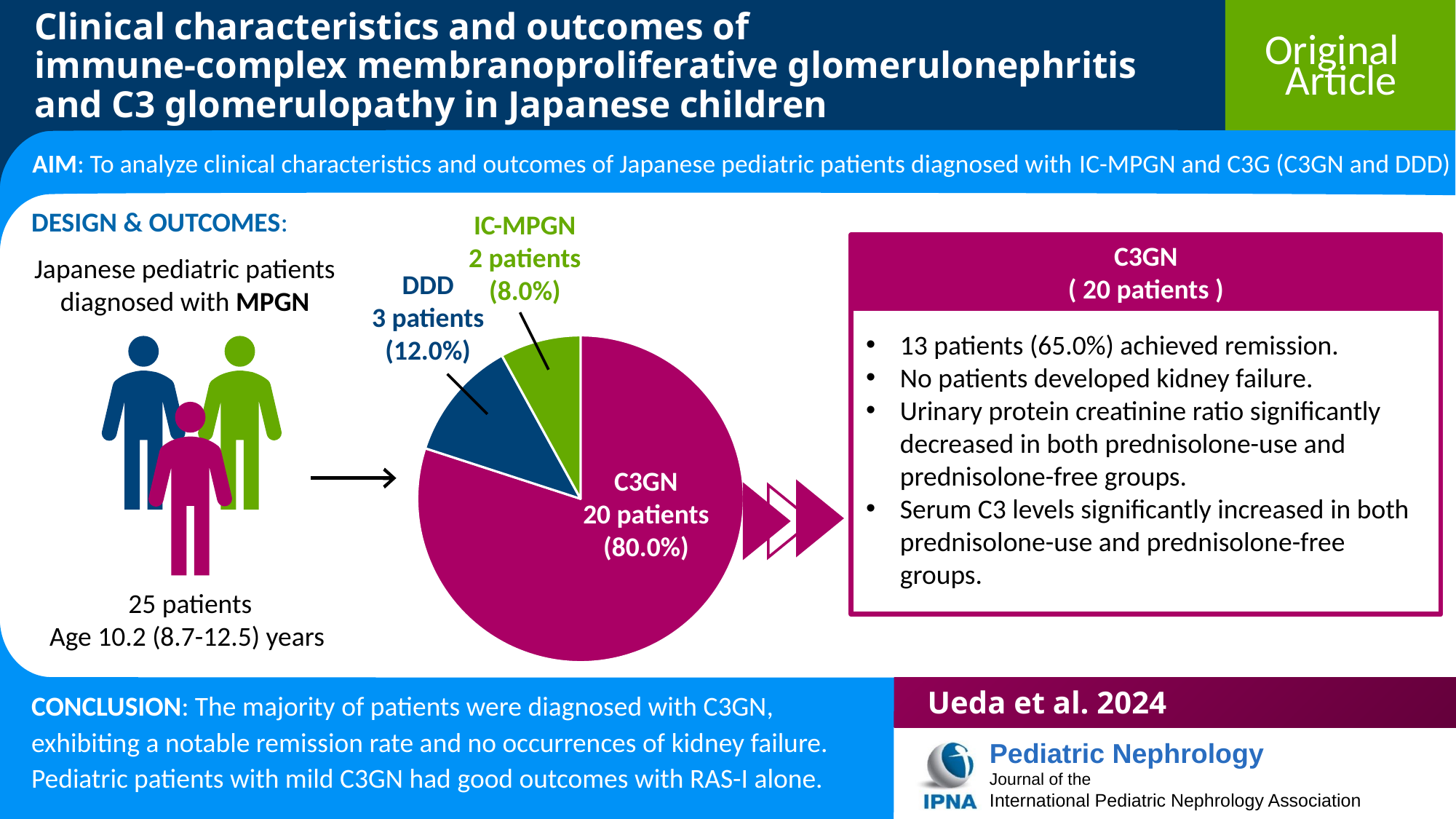

Clinical characteristics and outcomes of
immune-complex membranoproliferative glomerulonephritis
and C3 glomerulopathy in Japanese children
AIM: To analyze clinical characteristics and outcomes of Japanese pediatric patients diagnosed with IC-MPGN and C3G (C3GN and DDD)
DESIGN & OUTCOMES:
IC-MPGN
2 patients
(8.0%)
C3GN
( 20 patients )
Japanese pediatric patients
diagnosed with MPGN
DDD
3 patients
(12.0%)
13 patients (65.0%) achieved remission.
No patients developed kidney failure.
Urinary protein creatinine ratio significantly decreased in both prednisolone-use and prednisolone-free groups.
Serum C3 levels significantly increased in both prednisolone-use and prednisolone-free groups.
### Chart
| Category | 人数 |
|---|---|
| C3GN | 20.0 |
| DDD | 3.0 |
| IC-MPGN | 2.0 |
C3GN
20 patients
(80.0%)
25 patients
Age 10.2 (8.7-12.5) years
Ueda et al. 2024
CONCLUSION: The majority of patients were diagnosed with C3GN,
exhibiting a notable remission rate and no occurrences of kidney failure.
Pediatric patients with mild C3GN had good outcomes with RAS-I alone.
