# Supplementary material for: Clinical characteristics and outcomes of immune-complex membranoproliferative glomerulonephritis and C3 glomerulopathy in Japanese children
Source: Pediatr Nephrol. 2024 Apr 25;39(9):2679–89. doi: 10.1007/s00467-024-06377-7 (PMC11272671; doi:10.1007/s00467-024-06377-7)
Supplement: Supplementary file 2 — Supplementary file2 (DOCX 153 KB) [file 467_2024_6377_MOESM2_ESM.docx]

**Clinical characteristics of immune-complex membranoproliferative glomerulonephritis and C3 glomerulopathy in Japanese children**

Chika Ueda^1^, Tomoko Horinouchi^1^, Yuta Inoki^1^, Yuta Ichikawa^1^, Yu Tanaka^1^, Hideaki Kitakado^1^, Atsushi Kondo^1^, Nana Sakakibara^1^, China Nagano^1^, Tomohiko Yamamura^1^, Junya Fujimura^2^, Naohiro Kamiyoshi^3^, Shingo Ishimori^4^, Takeshi Ninchoji^5^, Hiroshi Kaito^6^, Yuko Shima^7^, Kazumoto Iijima^8 9^, Kandai Nozu^1^, Norishige Yoshikawa^10^

**Pediatric Nephrology**

**Corresponding author**

Tomoko Horinouchi MD, PhD

Department of Pediatrics, Kobe University Graduate School of Medicine, 7‑5‑1 Kusunoki‑cho, Chuo‑ku, 650‑0017, Kobe, Japan

Tel: +81-78-382-6090; Fax: +81-78-382-6099; E-mail: [tohori@med.kobe-u.ac.jp](mailto:tohori@med.kobe-u.ac.jp)

ORCID: https://orcid.org/0000-0003-1655-6030


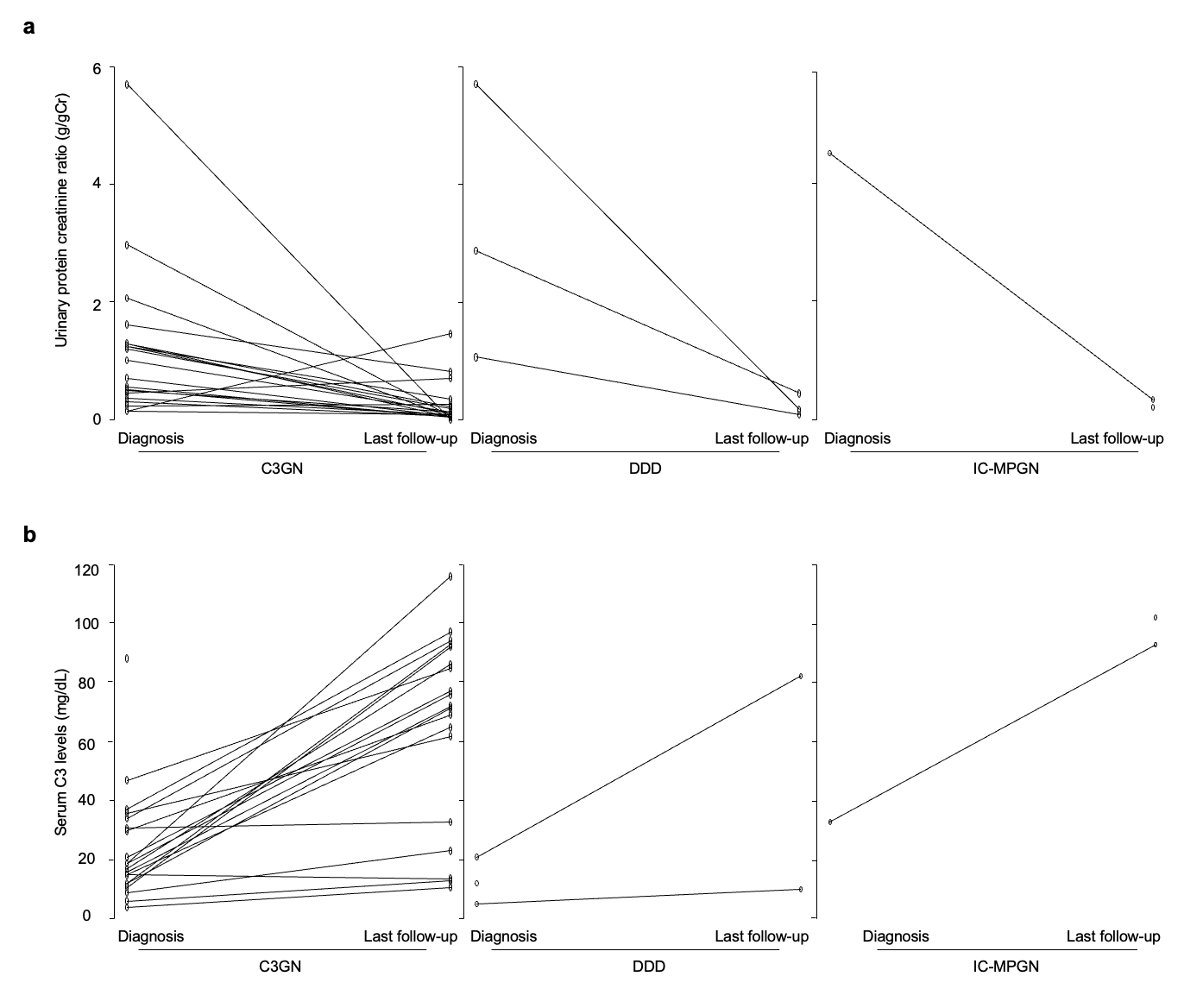
**Supplementary Fig. 1 Comparison of the data at diagnosis and at the last follow-up.** (a) Urinary protein creatinine ratio at the last follow-up tended to be decreased compared with that at the time of diagnosis in all groups. (b) Serum C3 levels at the last follow-up tended to be increased compared with those at the time of diagnosis in all groups

| Supplementary Table 1. Characteristics of normalized and non-normalized serum C3 cases in the C3GN group | | | |
| --- | --- | --- | --- |
|  | Normalized serum C3 (N=7) | Non-normalized serum C3 (N=12) | *P* value |
| Baseline |  |  |  |
| Age at diagnosis (year) | 8.7 (7.6-9.1) | 12.7 (11.4-14.2) | 0.02 |
| Serum Albumin (g/dL) | 3.8 (3.1-4.2) | 4.0 (3.8-4.2) | 0.44 |
| Nephrotic syndrome, N (%) | 2 (28.6) | 0 (0) | 0.12 |
| eGFR (ml/min/1.73m^2^) | 109.2 (87.8-123.8) | 131.1 (120.9-144.7) | 0.14 |
| eGFR < 90 ml/min/1.73m^2^, N (%) | 3 (42.9) | 1 (8.3) | 0.12 |
| Urinary protein creatine ratio (g/gCr) | 1.25 (0.78-2.51) | 0.60 (0.28-1.26) | 0.08 |
| Serum C3 (mg/dL) | 19.0 (14.5-35.5) | 15.6 (11.3-23.3) | 0.25 |
| Low C3 (≤ 80 mg/dL), N (%) | 7 (100.0) | 12 (100) | 1 |
| Histopathology |  |  |  |
| Crescents, N (%) | 2 (28.6) | 3 (25.0) | 1 |
| Endocapillary proliferation, N (%) | 2 (28.6) | 1 (8.3) | 0.52 |
| Global sclerosis, N (%) | 1 (14.3) | 0 (0) | 0.37 |
| Interstitial fibrosis, N (%) | 1 (14.3) | 4 (33.3) | 0.60 |
| Treatment |  |  |  |
| RAS-I, N (%) | 5 (71.4) | 12 (100) | 0.12 |
| Prednisolone, N (%) | 4 (57.1) | 7 (87.5) | 1 |
| MZR, MMF, N (%) | 0 (0) | 4 (33.3) | 0.25 |
| Last follow-up |  |  |  |
| Serum Albumin (g/dL) | 4.5 (4.2-4.9) | 4.3 (4.1-4.5) | 0.40 |
| eGFR (ml/min/1.73m^2^) | 104.6* (95.4-117.9) | 110.9 (106.3-122.5) | 0.44 |
| eGFR < 90 ml/min/1.73m^2^, N (%) | 1* (16.7) | 1 (8.3) | 1 |
| Urinary protein creatine ratio (g/gCr) | 0.04 (0.04-0.08) | 0.17 (0.08-0.27) | 0.02 |
| Remission | 7 (100.0) | 6 (50.0) | 0.04 |
| *Lack of data for one patient. | | | |

C3GN C3 glomerulonephritis, eGFR estimated glomerular filtration rate, RAS-I renin-angiotensin system inhibitor, MZR mizoribine, MMF mycophenolate mofetil
